# 

**Published:** 1859-01

**Authors:** 


### INDEX TO VOL. VI.

OF

### HALL'S JOURNAL OF HEALTH.

| | PAGE |
|---|---|
| Adulteration of Food | 191 |
| A Little Kills | 44 |
| Air Cure | 193, 194 |
| Autumnal Diseases | 212 |
| Amusements | 232 |
| Agriculture | 254 |
| Abuse of Medicine | 274 |
| Amenities of the Dining-Room | 258 |
| Anal Itchings | 289 |
| Animosities, Nursing | 289 |
| Buckwheat Cakes | 7 |
| Brain, Softening of | 126 |
| Broken Bones | 45 |
| Bread without Yeast | 149, 25, 73 |
| Bull-Dogs | 77 |
| Beautiful Old Age | 83 |
| Business Reverses | 101 |
| Baths and Bathing | 111 |
| Bottled Wrath | 128 |
| Black Bean Story | 146 |
| Build, where to | 175 |
| Bad Plans | 219 |
| Birds of the Wood | 266 |
| Bites and Stings | |
| Bad Breath | 275 |
| Burying Alive | 228 |
| Bostonian Complacency | 290 |
| Corn Bread | 25 |
| Consumption, 288, 290, 276, 158, 182, | 29 |
| Cellars | 31 |
| Careworn | 33 |
| Chair, Physiological | 75 |
| Children | 74, 81, 140, 288, 107 |
| Coolings | 130 |
| Constitutions Created | 146 |
| Clergymen, Death of | 215 |
| Corn Cobs Moral | 223 |
| Cramps | 225 |
| Cause of Disease | 237 |
| Car, R. R., Observations | 241 |
| Colds Cured | 283, 253 |
| Custom | 261 |
| Chimney Smoking | 277 |
| Contrast in Family Education | 288 |
| Corns Cured | 219 |
| Constipation | 284 |
| Dieting for Health | 27 |
| Disease and Crime | 33 |
| Declining, Premature | 46 |
| Depravity, Human | 61 |
| Daughters, about our | 74 |
| Drinking and Death | 117 |
| Difference, The | 147 |
| Death and Crime | 154 |
| Drowning | 176 |
| Doctors' Habits | 190 |
| Death Rate | 191 |
| Deadly Emanations | 195 |
| Disease and Suicide | 197 |
| Drinking Ice-Water | 216 |
| Dysentery | 217 |
| Dyspepsia and Drunkenness | 229 |
| Dining-Room Amenities | 258 |
| Encouragement | 44 |
| Eating, Object of | 85 |
| Emanations, Deadly | 195 |
| Eyes, Care of | 228 |
| Eating and Drinking | 280 |
| Experience, My own | 279 |
| Flannel Wearing | 10 |
| Fraternization Editorial | 104, 48 |
| Fever and Ague Prevented | 72 |
| Frost-Work | 90 |
| Fanaticism | 132 |
| Filth and Health | 181 |
| Food Adulteration | 191 |
| Food, Preservation of | 211 |
| Fuel | 221 |
| "Friends," Society of | 231 |
| Fun | 238 |
| Feet | 265 |
| Fire on the Hearth | 287 |
| Finger Nails | 291 |
| Growlers | 17 |
| Grave of Hope | 101 |
| Hair, the Human | 271, 11 |

| | PAGE |
|---|---|
| Health and Duty | 64 |
| Hotels | 74 |
| Heart Disease | 87 |
| Hope, the Grave of | 101 |
| Human Manufactory | 170 |
| Habitations Unhealthful | 173 |
| House Warming | 19 |
| Human Mortality | 183 |
| Howard the Philanthropist | 216 |
| Instinct | 49 |
| Insurance, Life | 53 |
| Inconsiderations | 134 |
| Impure Air | 193 |
| Inadvertences | 196 |
| Ice-Water Drinking | 138, 216 |
| Innocent Amusements | 232 |
| Intemperance of Old | 290, 234 |
| Insanity | 164, 197, 243 |
| India-Rubber Shoes | 250 |
| Impracticable Persons | 272 |
| Just About Right | 216 |
| Living Long | 24, 84, 135, 249 |
| Live Usefully | 41 |
| Locating for Life | 45 |
| Life Insurance | 53 |
| Liquor Drinking | 118 |
| Lunacy | 164 |
| Loose Bowels | 171 |
| Lightning Stroke | 178 |
| Life, Occupations of | 180 |
| Life, its Philosophy | 185 |
| Medicating One's Self | 18 |
| Medicine and Science | 58, 274 |
| Moral Nutriment | 69 |
| Milk | 92 |
| Music Healthful | 123, 141 |
| Mortalities | 183, 189, 215 |
| Marriage Contract | 216 |
| Medication, the Best | 241 |
| Measles | 251 |
| Morals of Sickness | 255 |
| Nature and Revelation | 47 |
| Nutriment Moral | 69 |
| Nose-hairs, etc | 209 |
| Nine Nevers | 237 |
| Object of Eating | 85 |
| Occupations of Great Men | 143 |
| Old People, Young | 144 |
| Occupations of Life | 180 |
| Pork as Food | 8 |
| Poverty, Disease, and Crime | 35 |
| Premature Decline | 46 |
| Physical Education | 68 |
| Poverty, Sympathize with | 80 |
| Pure Food | 120 |
| Papered Rooms | 175 |
| Populatory Calculations | 189 |
| Preservation of Food | 211 |
| Poisonous Bites and Stings | 233 |
| Pouting | 272 |

| | PAGE |
|---|---|
| Quackery Unmasked | [illegible]6 |
| Quintuple Alliance | [illegible]4 |
| Quenching Thirst | [illegible]7 |
| Reason and Instinct | 19 |
| Religious Daily Newspaper | 88 |
| Rat Riddance | 187 |
| Returning to Town | 263 |
| Rules for Winter | 282 |
| Self-Medication | 18 |
| Softening of the Brain | 26 |
| Suicidal Women | 40 |
| Science of Medicine | 58 |
| Student, Health | 73 |
| Sorrowing Poverty | 80 |
| Sores | 86 |
| Sleeping Together | 97 |
| Surfeits Cured | 99 |
| Spring Diseases | 106, 119 |
| Sabbath, Keeping the | 112, 207, 235 |
| Sudden Death | 113 |
| Stupidity | 122 |
| Summer Excursions | 125, 263 |
| Seasonable Hints | 144 |
| Summer Sours | 147 |
| Summer Resorts | 163 |
| Sleeping in Church | 171, 181 |
| Sydenham's Habits | 190 |
| Suicide | 197, 327 |
| Scrofula | 245 |
| Shoes, India-Rubber | 250 |
| Sickness and its Morals | 255 |
| Sunday Dinners | 259 |
| Sunshine, Its Healthfulness | 64 |
| Smoky Chimney | 277 |
| True Temperance | 50 |
| Thankful Be | 67 |
| Temperament Differences | 109 |
| Thirst Quenched | 117 |
| Town and Country | 139 |
| Tomatoes | 218 |
| Take Life Easy | 257 |
| Teachers of our Children | 269 |
| Taking Cold | 283 |
| Uses of Ice | 138 |
| Unhealthful Habitations | 173 |
| Use of Sunshine | 175 |
| Wearing Flannel | 10 |
| Warming Houses and Churches | 19, 42 |
| Warts | 71 |
| Well and Spring Cleaning | 72 |
| Weakly Youths | 81 |
| Walk Softly | 82 |
| Well Done | 111 |
| Why Children Die | 140 |
| Where to Build | 175 |
| Wearing Rubber Shoes | 250 |
| World's Workers | 278 |
| Winter Rules | 282 |
| Young Old People | 144 |
| Youths, Sickly | 81 |

